# Unlocking the potential of embryos: insight of systematic review and meta-analysis into laser-assisted hatching’s role in conquering recurrent implantation failure

**DOI:** 10.3389/frph.2025.1581529

**Published:** 2025-05-09

**Authors:** Tingting Du, Yang Ma, Zanche Huang, Xiaoxuan Zhao, Qin Zhang, Ning Ren

**Affiliations:** Department of Medical TCM Gynecology, Hangzhou TCM Hospital of Zhejiang Chinese Medical University (Hangzhou Hospital of Traditional Chinese Medicine), Hangzhou, Zhejiang, China

**Keywords:** laser assisted hatching, recurrent implantation failure, implantation rate, clinical pregnancy rate, abortion rate

## Abstract

**Introduction:**

The journey of assisted reproductive technology (ART) for couples facing recurrent implantation failure (RIF) is fraught with emotional and physical challenges. RIF, often characterized by the failure of high-quality embryos to implant after multiple ART cycles, has directed attention towards interventions like laser-assisted hatching (LAH). However, discrepancies in the literature necessitate a comprehensive review of LAH's efficacy and safety.

**Materials and methods:**

Following a thorough search of PubMed, Embase, Cochrane, and Web of Science databases up to November 2023, retrospective studies or RCT were considered for inclusion. Summary effect sizes [odds ratio (OR) or risk ratio (RR) with 95% confidence interval (CI)] were calculated for each outcome.

**Results and conclusion:**

Eight studies comprising 2,634 patients were included. LAH significantly improved implantation rates (OR: 1.26, 95% CI: 1.05–1.51). Clinical pregnancy rates increased in patients who had fresh embryos transferred (OR: 1.29, 95% CI: 1.05–1.58). Notably, LAH was associated with higher miscarriage rates in frozen embryo transfers (OR: 1.45, 95% CI: 1.04–2.02). No significant increase in ectopic or multiple pregnancy rates was observed. For patients with RIF, especially older women, LAH presents a potential avenue to improve implantation. Its impact on clinical pregnancy rates is less substantial. However, its impact on final live birth rates and the increased miscarriage risk in frozen transfers necessitate a cautious and individualized approach. The technique's safety, while generally upheld, requires careful application and consideration of the specific challenges RIF patients face.

**Systematic Review Registration:** https://www.crd.york.ac.uk/PROSPERO/view/CRD42024497329, PROSPERO (CRD42024497329).

## Introduction

The journey of assisted reproductive technology (ART) is marked by both remarkable successes and notable challenges (1), among which repeated implantation failure (RIF) stands out as a particularly distressing obstacle for many aspiring parents. According to the current consensus, RIF is generally defined as the absence of clinical pregnancy after three or more euploid embryos (2). Based on a meta-analysis of 119 articles, Polanski et al. proposed that RIF should be defined as failure to achieve clinical pregnancy after 2 consecutive embryo transfer failures (1). We also found that most of the clinical studies to determine the efficacy of LAH interventions have been conducted on patients with ≥2 embryo transfer failures. Therefore, in this meta-analysis, we defined RIF as failure to obtain a clinical pregnancy after at least 2 ET, and clinical pregnancy was defined as ultrasonographic confirmation of survival after 20 weeks of gestational age, consistent with the definition of miscarriage. RIF not only represents a significant clinical dilemma but also inflicts profound emotional distress on affected couples (3). This condition, affecting a considerable proportion of couples undergoing ART, has been a focus of extensive research and debate within the reproductive medicine community.

The pathophysiology of RIF is multifaceted, involving factors such as embryonic aneuploidy, uterine receptivity issues, and immunological factors (4). However, a particular area of interest has been the potential role of the zona pellucida (ZP), the outer glycoprotein layer of the embryo, in impeding embryo hatching and subsequent implantation (5). This has led to the exploration of techniques to facilitate embryo hatching, among which laser-assisted hatching (LAH) has emerged as a promising intervention.

LAH involves the use of a highly focused infrared laser to precisely thin or create a breach in the ZP, with the aim of facilitating the embryo's escape, a critical step for successful implantation (6, 7). Initial studies into LAH have shown encouraging results, particularly in enhancing implantation rates in challenging cases such as those of older women or patients with a history of RIF (8). However, the literature presents a somewhat conflicting view regarding the efficacy of LAH. While several studies advocate for the benefits of LAH in improving clinical outcomes, others report no significant improvement, bringing into question the universal applicability and effectiveness of this technique (9, 10).

This discrepancy in research findings underscores the need for a comprehensive and systematic review of existing studies. Therefore, this meta-analysis aims to synthesize the available data to provide a clearer understanding of the role of LAH in improving outcomes for RIF patients. By assessing the efficacy and safety of LAH, this study intends to offer valuable insights into its potential as a tool to enhance ART success rates, especially in the context of RIF.

## Materials and methods

### Search strategy

Two investigators independently searched PubMed, Embase, Cochrane and Web of Science from inception to Nov. 22, 2023. All retrievals utilized Medical Subject Headings (MeSH) and free words; the complete search string is detailed in Supplementary Method S1–S4. We further reviewed the relevant professional vocabulary of the references in the thematic review to ensure the inclusion of all pertinent search terms. The search term was ‘IVF’ OR ‘ICSI’, in combination with ‘recurrent implantation failure’, in combination with ‘laser assisted hatching’, with no restriction of countries, region, race, and language.

### Inclusion and exclusion criteria

Registration adhered to the Preferred Reporting Program for Systematic Review and Meta-Analysis (PRISMA) guidelines. The protocol of our study has been registered in PROSPERO (CRD 42024497329).The inclusion criteria were as follows: (1) Patients with RIF, while RIF was defined in this study as patients had experienced at least two or more failed *in vitro* fertilization (IVF) or intracytoplasmic sperm injection (ICSI) attempts; (2) Use of LAH in the intervention group and mechanical hatching (MAH), chemical hatching (CAH), or non-assisted hatching in the control group; (3) Provision of odds ratio (OR) and 95% confidence interval (CI) data on implantation rate, clinical pregnancy rate, miscarriage rate, live birth rate, and risk ratio (RR) with 95% CI on multiple pregnancy rate, ectopic pregnancy rate, and adverse pregnancy events;

The exclusion criteria were as follows: (1) Animal or *in vitro* test; (2) Article type: letters, review, meta-analysis, comment, case report, conference abstract, editorials, expert opinions, etc. (3) Studies not employing LAH in the intervention group or utilizing it in the control group. Two investigators established the inclusion and exclusion criteria, while a third reviewer provided assistance in achieving consensus when necessary.

### Data extraction

The following characteristic information of the included studies was recorded: (1) Study characteristics: first author, publication time, repeated failures of transfer, sample size, age range, regimen, embryo state, embryo per transfer; (2) Study outcomes: clinical pregnancy rate, abortion rate, implantation rate, risky events, live birth rate. To make sure that no information was omitted, we also checked supplement materials of each clinical trial.

### Quality assessment

A tool named Risk of Bias In Non-randomized Studies-of Interventions (ROBINS-I), employing a weighted Cohen's kappa coefficient (κ), was used to measure agreement, with any discrepancies between the two investigators being resolved by consensus. Each study was defined as ‘low risk’, ‘moderate risk’, ‘serious risk’, ‘critical risk’ or ‘no information’ respectively by considering the following characteristics covering bias due to confounding; bias in selection of participants; bias in classification of interventions; bias due to deviations from intended interventions; bias due to missing data; bias in measurement of outcomes; and bias in selection of the reported result (Supplementary Table S1).

### Statistical analysis

Statistical analyses of study outcomes were conducted using Stata 15. For the included studies, the dichotomous data results for each of the studies eligible for meta-analysis were expressed as an odds ratio (OR) with 95% confidence intervals (CI), and as a relative risk (RR) with 95% CI for risk events. The Chi-square Q test and *I*^2^ statistic was used to detect statistical heterogeneity. If heterogeneity was observed between studies (*p* < 0.10, *I*^2^ > 50%), a random-effects model would be employed for the combined analysis. Otherwise, the fixed-effects model was used for the pooled results. Furthermore, subgroup analysis was implemented to identify the factors contributing risk of bias; sensitivity analysis was also performed by sequentially excluding each included trial. Funnel plots, Begg's and Egger's tests were also used to examine potential publication bias. To evaluate the power, *p* < 0.05 was considered statistically significant.

## Result

### Literature search and patients characteristics

**Figure 1:**
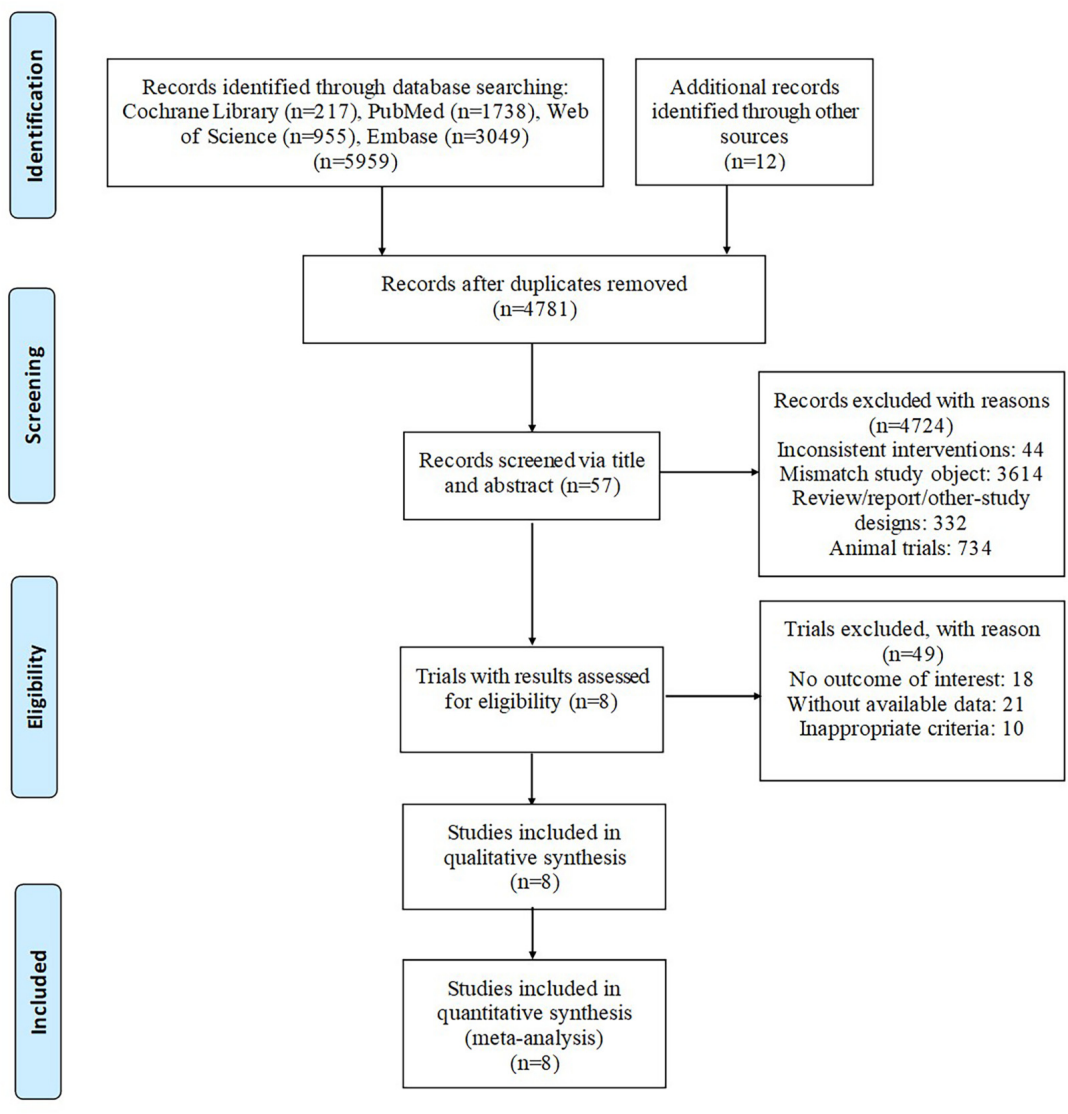
Articles retrieved nnand assessed for eligibility. After screening process, 8 articles met the including criteria and were included in ultimate analysis.

According to the proposed search strategy, 5,959 publications were retrieved. After removing duplicates, 4,781 articles remained. Then, by screening title and abstract only, 57 articles were assessed for eligibility. Finally, after assessing the full text of the remaining articles, 8 studies (11–18) were included in qualitative synthesis. The specific selection steps are summarized in Figure 1.

Of the eligible eight studies, four studies were randomized controlled trials (RCTs), while the other four were retrospective studies. A total of 2,634 patients were enrolled in our study. The age of participants ranged from 28 to 42 years across seven studies, only one remaining study did not report the age. Two studies used Zona thinning as an intervention measure, the other six studies used Zona drilling. The regimen of control groups included no assisted hatching and chemical hatching. Six studies reported embryo per transfer. The main characteristics and results are shown in Table 1.

**Table 1:** Characteristic of included studies.

Study	Study type	Group	Mean ± SD age (years)	Sample size	Embryo per transfer (Mean ± SD)
Petersen et al. (18)	RCT	I	35.7 ± 3.8	40	3.0 ± 0.9
		C	35.3 ± 5.1	40	2.9 ± 0.8
Lee et al. (17)	RCT	I	37.6 ± 4.2	48	3.1 ± 1.1
		C	36.6 ± 4.1	79	3.1 ± 1.1
Debrock et al. (16)	Retrospective study	I	33.7 ± 4.5	53	2.02 ± 0.31
		C	33.8 ± 4.1	86	1.93 ± 0.48
Choi et al. (15)	RCT	I	NA	22	2.6 ± 0.1
		C	NA	20	2.7 ± 0.1
Lu et al. (14)	Retrospective study	I	32.0 ± 3.4	225	2.1 ± 0.6
		C	31.6 ± 3.1	190	2.2 ± 0.4
Artar et al. (13)	Retrospective study	I	32.8 ± 5.2	100	NA
		C	34.7 ± 5.2	215	NA
Pan et al. (12)	Retrospective study	I	30.6 ± 2.6	390	1.94 ± 0.31
		C	30.9 ± 2.8	534	1.97 ± 0.24
Curfs et al. (11)	RCT	I	34.2 ± 4.3	297	NA
		C	34.3 ± 4.4	295	NA

Abbreviations: RCT, randomized controlled trial; I, intervention; C, comparator; AH, assisted hatching.

### Quality assessment

Five studies (Petersen et al (18), Debrock et al. (16), Lu et al. (14), Artar et al. (13), Pan et al. (12)) was rated as moderate based on the ROBINS-I tool (Supplementary Table S1). The left three studies (Lee et al. (17), Choi et al. (15), Curfs et al. (11)) was labelled as low. Supplementary Table S1 demonstrates moderate to high inter-rater agreement for risk of bias assessments, with *κ* values ranging from 0.60 to 1.00 across domains.

### Efficacy

#### Implantation rate

**Figure 2:**
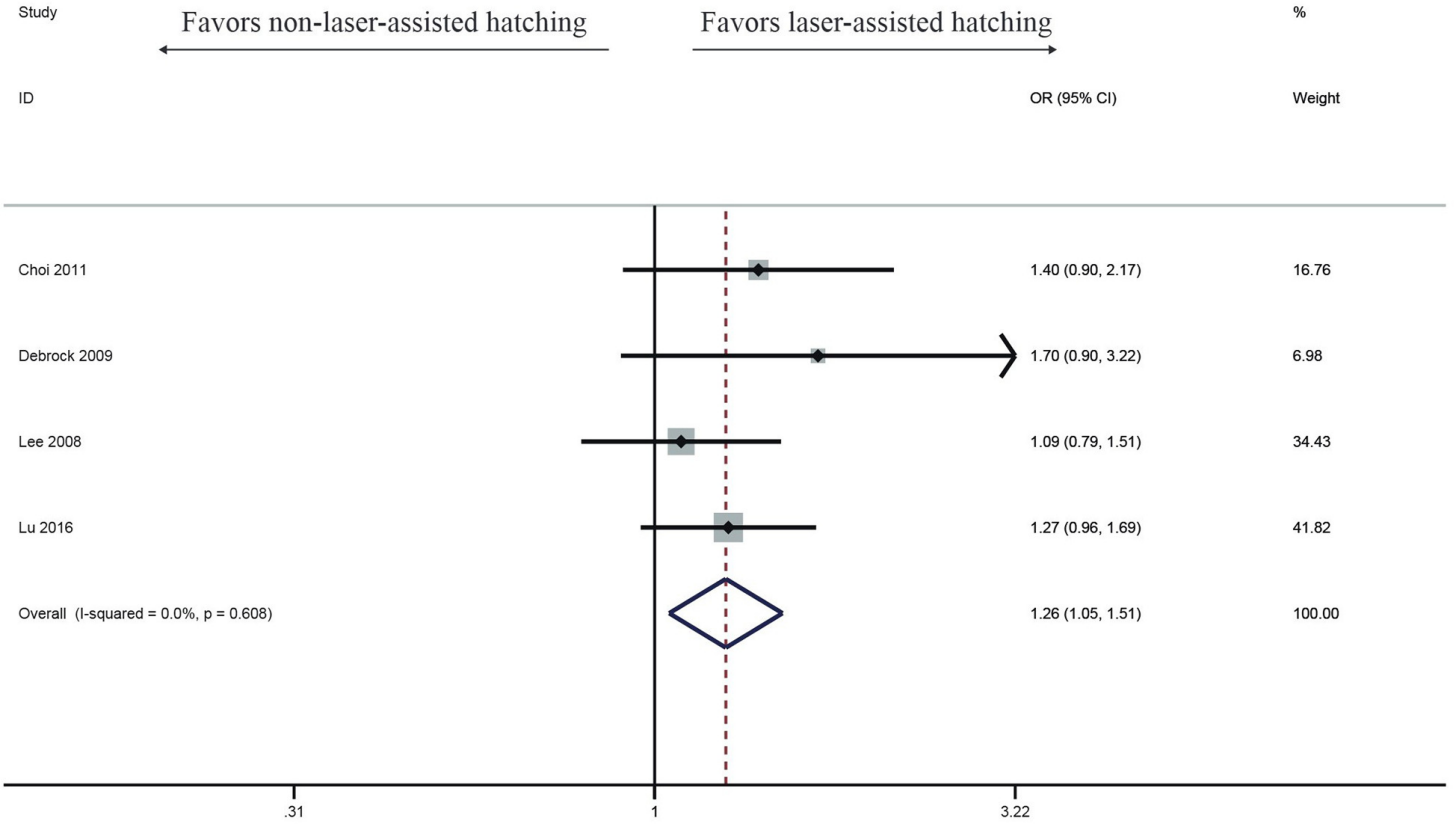
Forest plot of the meta-analysis of implantation rate.

Four studies investigated the impact of LAH on Implantation rate. Compared with the control group, the success rate of implantation rate in the LAH group (OR: 1.26, 95% CI: 1.05–1.51) (Figure 2) significantly increased, no heterogeneity was found (*I*^2^ = 0%, *p* = 0.608).

#### Clinical pregnancy rate

**Figure 3:**
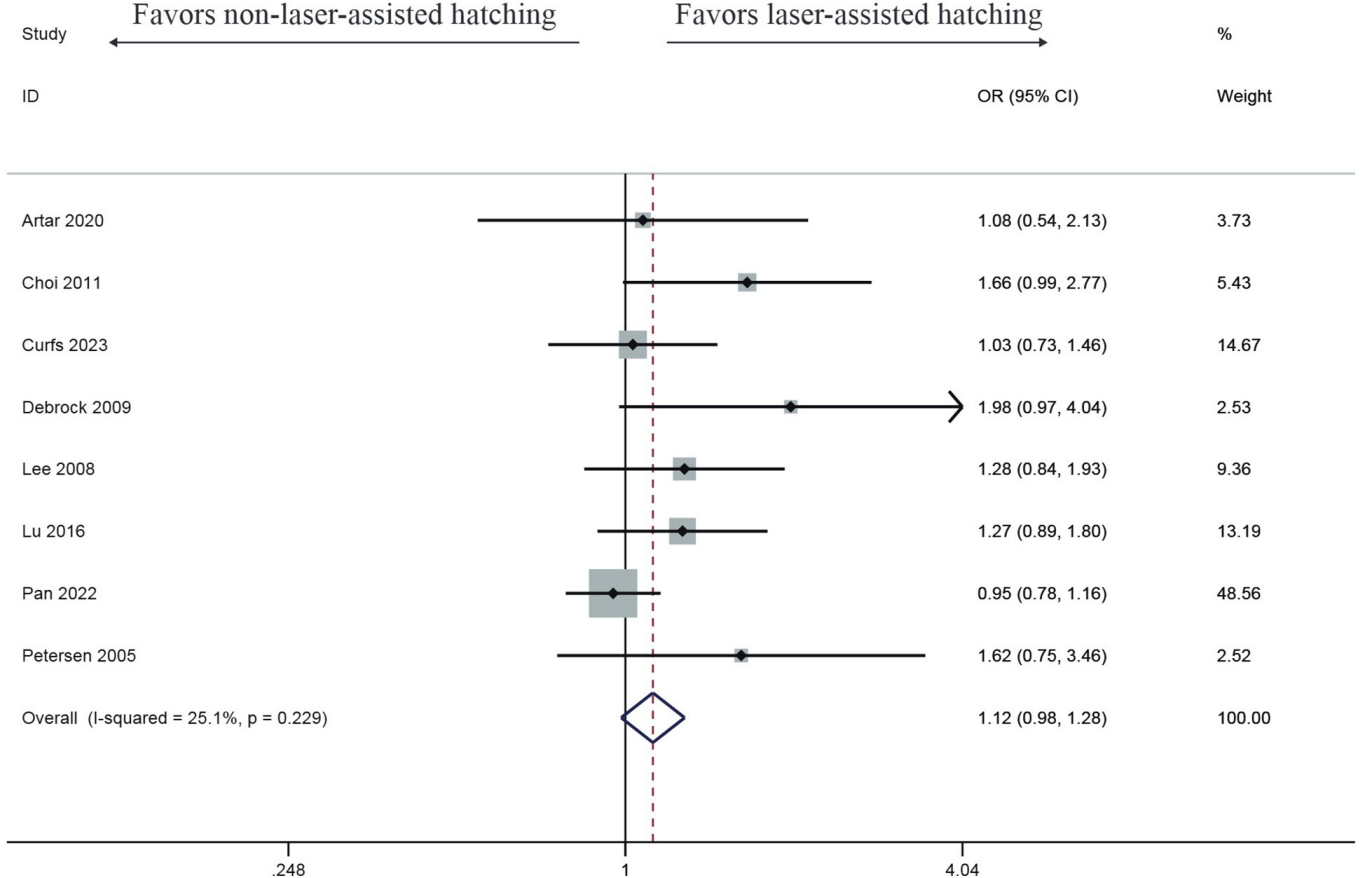
Forest plot of the meta-analysis of clinical pregnancy rate.

Eight studies observed that the clinical pregnancy rate in the LAH group was improved, but there was no significant difference (OR: 1.12, 95% CI: 0.98–1.28) (Figure 3), with no heterogeneity (*I*^2^ = 25.1%, *p* = 0.229).

#### Abortion rate

**Figure 4:**
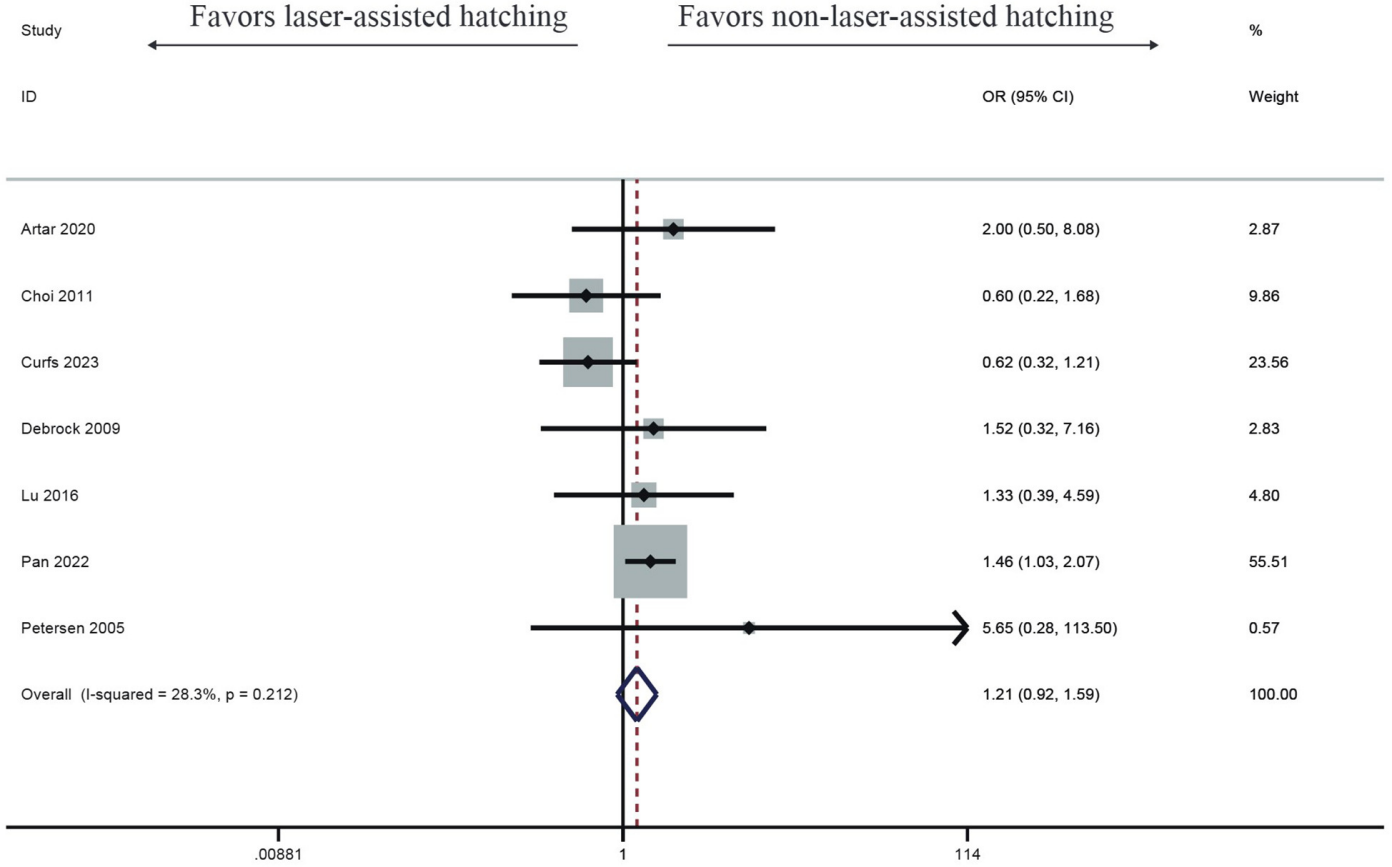
Forest plot of the meta-analysis of abortion rate.

Seven studies investigated the impact of LAH on abortion rate. Compared with the control group, the LAH group showed a higher miscarriage rate, but there was no significant difference (OR: 1.21, 95% CI: 0.92–1.59) (Figure 4), with no heterogeneity (*I*^2^ = 28.3%, *p* = 0.212).

#### Live birth rate

**Figure 5:**
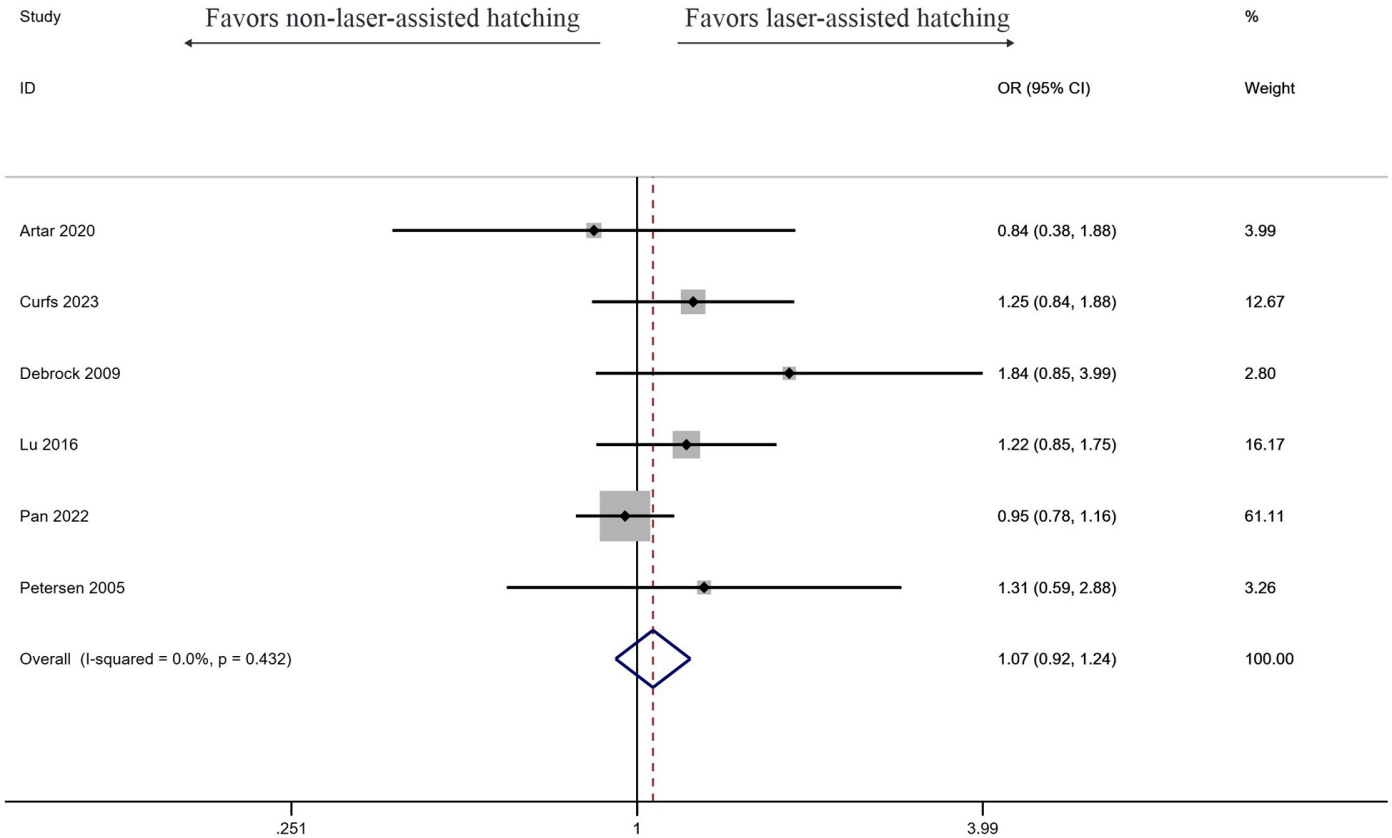
Forest plot of the meta-analysis of live birth rate.

Six studies investigated the effect of LAH on live birth rate, and the results showed LAH brought significant increase (OR: 1.07, 95% CI: 0.92–1.24) (Figure 5). But the result showed no significant difference, and no heterogeneity was found (*I*^2^ = 0%, *p* = 0.432).

### Subgroup analysis

Subgroup analysis of efficacy was performed across predefined subgroups (Table 2). A benefit in implantation rate was observed among patients aged ≥38 years (OR: 2.10, 95% CI: 1.20–3.69). In contrast, patients under 38 years failed to achieve an improved implantation rate (OR: 1.00, 95% CI: 0.74–1.36) (Supplementary Figure S6). Moreover, a trend favoring LAH was evident in clinical pregnancy rates for patients with fresh embryo transfers (OR: 1.29, 95% CI: 1.05–1.58); however, no improvement was observed in patients with frozen embryo transfers (OR: 1.02, 95% CI: 0.86–1.21) (Supplementary Figure S7). Additionally, LAH showed no significant difference inlive birth rates for patients with either fresh (OR: 1.26, 95% CI: 0.93–1.70) or frozen embryos transfers (OR: 1.01, 95% CI: 0.85–1.20) (Supplementary Figure S8). Besides, patients carried LAH were more likely to experience miscarriage with frozen embryo transfers (OR: 1.45, 95% CI: 1.04–2.02), yet less likely with fresh embryo transfers, though this difference was not statistically significant (OR: 0.85, 95% CI: 0.53–1.37) (Supplementary Figure S9).

**Table 2:** Selected subgroup analysis of pregnancy outcomes.

Variables		Implantation rate	Clinical pregnancy rate	Live birth rate	Abortion rate
		OR(95% CI)	OR(95% CI)	OR(95% CI)	OR(95% CI)
Embryonic status	Fresh	/	1.29 (1.05–1.58)	1.26 (0.93–1.70)	0.85 (0.53–1.37)
	Frozen	/	1.02 (0.86–1.21)	1.01 (0.85–1.20)	1.45 (1.04–2.02)
Embryonic phase	Cleavage-stage	/	/	/	/
	Blastocyst	/	/	/	/
Age	≥38	2.10 (1.20–3.69)	/	/	/
	<38	1.00 (0.74–1.36)	/	/	/

### Safety

Only two studies have reported the rates of ectopic pregnancy and multiple pregnancies (Table 3). The study observed that the ectopic pregnancy rate in the LAH group was lower than that in the control group, but there was no statistically significant difference (RR: 0.68, 95% CI: 0.36–1.26) (Supplementary Figure S10A). Meanwhile, patients in the LAH group tended to have a lower multiple pregnancy rate, which was also not statistically significant (RR: 0.82, 95% CI: 0.65–1.02) (Supplementary Figure S10B).

**Table 3:** Treatment-related common high-risk pregnancy events in this meta-analysis.

High-risk pregnancy events	RR(95% CI)
Ectopic	0.68 (0.36–1.26)
Multiple	0.82 (0.65–1.02)

### Publication bias and sensitivity analysis

Begg's Test and Egger's test were carried out to access publication bias in this study. No publication bias was found with Begg's test and Egger's test for implantation rate (Egger's test *p* = 0.241, Begg's Test *p* = 0.308) (Supplementary Figures S2A–C), live birth rate (Egger's test *p* = 0.18, Begg's Test *p* = 1.0) (Supplementary Figures S4A–C), abortion rate (Egger's test *p* = 0.978, Begg's Test *p* = 0.368) (Supplementary Figures S5A–C). However, there was slight publication bias in clinical pregnancy rate (Egger's test *p* = 0.012, Begg's Test *p* = 0.063) (Supplementary Figures S3A–C).

Sensitivity analysis was conducted to evaluate the impact of individual research findings on overall outcomes (Supplementary Figures S1A–D). Most of the literature show significant statistical changes in the overall results after exclusion, which indicated a certain degree of bias in the meta-analysis.

## Discussion

In our study, outcomes from eight studies were critically analyzed and compared. Consistent with the meta-analysis by Zeng et al., our research confirms that laser-assisted hatching (LAH) enhances clinical pregnancy and implantation rates, indicating its potential in improving early reproductive outcomes. This partially overlaps with and contradicts our results, as we found that implantation rates were more significant in older women and clinical pregnancy was related to the transfer of fresh embryos. Furthermore, both studies observed that LAH does not significantly increase live birth rates, suggesting its benefits may be limited to the initial stages of pregnancy. However, LAH is also associated with an increased likelihood of miscarriage in frozen embryo transfers. Additionally, both studies highlight the increased risk of multiple pregnancies associated with LAH, underscoring the need for meticulous patient counseling, although this risk was not statistically significant in our study.

Patients with RIF often struggle with embryos that fail to implant after multiple cycles. With advancing maternal age, the zona pellucida tends to become thicker and harder. This change can impede the natural hatching process, and makes it more difficult for embryos to implant in the uterine lining (19, 20). LAH can overcome this barrier, thereby improving the odds of successful implantation and clinical pregnancy (21, 22). And for successful implantation, the timing of embryo hatching needs to be synchronized with the window of endometrial receptivity. LAH might improve the synchronization between the embryo's readiness to implant and the endometrial receptivity, a crucial factor for patients with a history of implantation failures. This synchronization is particularly beneficial for older women whose endometrial receptivity might be more variable (19, 23). Recent studies and meta-analyses have also provided more insights into the efficacy of LAH, especially in the context of older women. These studies have started to elucidate the specific scenarios in which LAH might be most beneficial, including cases involving older women with previous implantation failures (24, 25). In addition, we strongly advocate for the adoption of more stringent criteria in future research and recommend further validation of LAH's efficacy specifically in rigorously defined RIF populations, with particular emphasis on long-term pregnancy outcomes in advanced-age cohorts.

Despite the overall analysis not showing a significant improvement in clinical pregnancy rates, it's noteworthy that a distinct improvement was observed in cases with fresh embryos, while no statistical difference was found for frozen embryos. For fresh embryo transfers, LAH may better synchronize the timing between embryo readiness and endometrial receptivity. The fresh transfer cycle might present a more synchronized environment for the embryo to implant, and the LAH's facilitation of hatching could align well with this natural timeline (23). Fresh embryos are generally considered to be of better quality than frozen ones as they have not been subjected to the potential stresses of freezing and thawing. The better initial quality of fresh embryos might mean that they are more capable of benefiting from the improved implantation conditions facilitated by LAH (25). As for frozen embryos, they would undergo stress during the freezing and thawing processes, which can lead to sub-lethal damages that aren't immediately apparent but might affect the embryo's ability to implant and develop, even after successful hatching facilitated by LAH (21). What's more, the cycles for frozen embryo transfers often involve hormone replacement therapy to prepare the endometrium, which might not mimic the natural cycle's endometrial receptivity as closely as fresh transfer cycles do. This could mean that even if the embryo is ready to implant due to LAH, the endometrial environment might not be optimally receptive (20).

As discussed above, the initial damage brought by the process of freezing and thawing imposes stress on the cells of the embryo (26, 27), and harder zona pellucida of frozen-thawed embryos, though thinned or driller by LAH, would affect the embryo's ability to expand and grow post-implantation, potentially leading to an increased risk of miscarriage (21, 28). Often, the best quality embryos are selected for fresh transfer, while the remaining viable ones are frozen for future use. This selection bias might inherently lead to a higher miscarriage rate in frozen embryo transfers as they might be of slightly lower quality or resilience compared to those chosen for fresh transfer (28, 29). Also, altered microenvironment by preparation protocols for frozen embryo transfers might also affect the success of implantation and ongoing pregnancy (19, 29). Besides, embryos with chromosomal abnormalities are more likely to miscarry. While LAH may assist in implantation, it doesn’t rectify underlying genetic issues that may be more prevalent in embryos that have undergone the stress of freezing and thawing (30, 31). While the observed increase in miscarriage rates for frozen embryos as compared to fresh embryos post-LAH is concerning, it's important to consider the multifaceted nature of these findings. Freezing embryos is a crucial aspect of ART, particularly for fertility preservation and reducing the risk of ovarian hyperstimulation syndrome (OHSS), the potential for increased miscarriage rates needs careful consideration (23, 32).

The lack of a statistically significant difference in final live birth rates with the use of LAH suggests that while LAH may influence earlier stages of embryo development or implantation, its impact does not extend to increasing the overall likelihood of a successful birth. Patients with RIF represent a heterogeneous group with various underlying causes contributing to their implantation failures. The inherent quality of the embryo is a crucial determinant of successful live birth. While LAH may assist in the hatching process, it does not enhance the genetic quality or the developmental potential of the embryo, which are critical for a successful full-term pregnancy (22). LAH is primarily a mechanical intervention designed to assist the embryo in breaking through the zona pellucida. While this may facilitate implantation, it does not necessarily improve the embryo's ability to develop and grow throughout the entire pregnancy (19, 20). Pregnancy and live birth are multistage processes influenced by various factors beyond initial embryo implantation. Factors such as genetic embryo viability (33, 34), maternal health (35, 36), uterine environment (37), and other unforeseen complications (37–39) during pregnancy all play significant roles in determining successful live birth (Shapiro et al., 2018). And only limited data in this paper was included in our paper, which may make the results less comprehensive, particularly large, well-designed randomized controlled trials, is necessary to clarify the role of LAH in influencing final live birth rates. Future studies should aim to identify specific patient subgroups that may benefit most from LAH (22, 24).

### Safety

The safety of LAH can be assessed by looking at associated risks like ectopic pregnancy and multiple births. While LAH improves implantation and pregnancy rates, it may also increase the chances of multiple pregnancies, which carry higher risks for both the mother and babies. However, current data, including from meta-analyses, doesn't suggest a significant increase in ectopic pregnancy rates. While LAH is generally considered safe, there is a potential risk of physical damage to the embryo if not performed correctly. For RIF patients, any additional risk needs careful consideration given their already challenging path to successful pregnancy. Ongoing research is needed to ensure the long-term safety of LAH, including any potential impacts on offspring health.

### Strengths and limitation

Our study's strength lies in its focused analysis on a specific demographic (women with RIF), providing valuable insights into the efficacy of LAH in this group. The study's focus on subgroups, such as older women and different embryo types (fresh vs. frozen), provides valuable insights into where LAH might be most effective. Despite its strengths, there are some weaknesses in our meta-analysis. The study's limitation is the small sample size, large RCT is needed in the future. Besides, potential publication bias was spotted in clinical pregnancy rate. The included studies span more than a decade, from 2005 to 2023. The development of assisted reproduction technologies over this decade is rapid, and the impact of technological differences on outcomes should be considered. However, our inclusion and expulsion process are rigorous, and the biphasic evaluation is reliable.

## Conclusion

For patients with RIF, LAH improves implantation rates in RIF patients and shows a trend towards improved clinical pregnancy rates, albeit without affecting live birth rates. The technique's safety profile is substantiated by the absence of increased ectopic or multiple pregnancies. However, the higher abortion rate with frozen transfers warrants cautious patient selection and personalized treatment strategies. Ongoing research and a comprehensive treatment strategy are essential for optimizing outcomes for these patients.

## Data Availability

The original contributions presented in the study are included in the article/Supplementary Material, further inquiries can be directed to the corresponding authors.

## References

[1] Farquhar C Marjoribanks J. Assisted reproductive technology: an overview of cochrane reviews. Cochrane Database Syst Rev. (2018) 8(8):Cd010537. 10.1002/14651858.CD010537.pub5 30117155 PMC6953328

[2] Pirtea P Cedars MI Devine K Ata B Franasiak J Racowsky C Recurrent implantation failure: reality or a statistical mirage?: consensus statement from the July 1, 2022 lugano workshop on recurrent implantation failure. Fertil Steril. (2023) 120(1):45–59. 10.1016/j.fertnstert.2023.02.014 36822566

[3] Coughlan C Ledger W Wang Q Liu F Demirol A Gurgan T Recurrent implantation failure: definition and management. Reprod Biomed Online. (2014) 28(1):14–38. 10.1016/j.rbmo.2013.08.011 24269084

[4] Bashiri A Halper KI Orvieto R. Recurrent implantation failure-update overview on etiology, diagnosis, treatment and future directions. Reprod Biol Endocrinol. (2018) 16(1):121. 10.1186/s12958-018-0414-2 30518389 PMC6282265

[5] De Vos A Van Steirteghem A. Zona hardening, zona drilling and assisted hatching: new achievements in assisted reproduction. Cells Tissues Organs (Print). (2000) 166(2):220–7. 10.1159/000016734 10729729

[6] Xu W Zhang L Zhang L Jin Z Wu L Li S Laser-assisted hatching in lower grade cleavage stage embryos improves blastocyst formation: results from a retrospective study. J Ovarian Res. (2021) 14(1):94. 10.1186/s13048-021-00844-7 34261510 PMC8281458

[7] Uppangala S D’Souza F Pudakalakatti S Atreya HS Raval K Kalthur G Laser assisted zona hatching does not lead to immediate impairment in human embryo quality and metabolism. Syst Biol Reprod Med. (2016) 62(6):396–403. 10.1080/19396368.2016.1217952 27598006

[8] Hsieh YY Huang CC Cheng TC Chang CC Tsai HD Lee MS. Laser-assisted hatching of embryos is better than the chemical method for enhancing the pregnancy rate in women with advanced age. Fertil Steril. (2002) 78(1):179–82. 10.1016/s0015-0282(02)03172-2 12095510

[9] Practice Committee of the American Society for Reproductive Medicine, Practice Committee of the Society for Assisted Reproductive Technology. The role of assisted hatching in *in vitro* fertilization: a guideline. Fertil Steril. (2022) 117(6):1177–82. 10.1016/j.fertnstert.2022.02.020 35618358

[10] Maron DJ Hochman JS Reynolds HR Bangalore S O'Brien SM Boden WE Initial invasive or conservative strategy for stable coronary disease. N Engl J Med. (2020) 382(15):1395–407. 10.1056/NEJMoa1915922 32227755 PMC7263833

[11] Curfs M Cohlen BJ Slappendel EJ Schoot DC Derhaag JG van Golde RJT A multicentre double-blinded randomized controlled trial on the efficacy of laser-assisted hatching in patients with repeated implantation failure undergoing IVF or ICSI. Hum Reprod. (2023) 38(10):1952–60. 10.1093/humrep/dead173 37646072 PMC10546076

[12] Pan JP Liang SS Huang MY Zhao M Kong PC Liu YP Obstetric and neonatal outcomes after frozen-thawed embryos transfer with laser-assisted hatching: a retrospective cohort study. Arch Gynecol Obstet. (2022) 305(2):529–34. 10.1007/s00404-021-06153-0 34390385

[13] Artar I Karlı P Başbuğ A Başbuğ D Eyi EGY Doǧan M. Evaluation of effectiveness of laser assisted hatching pregnancy rates on fresh IVF/ICSI cycles. Gazi Med J. (2020) 31(4):537. 10.12996/gmj.2020.126

[14] Lu X Liu Y Cao X Liu SY Dong X. Laser-assisted hatching and clinical outcomes in frozen-thawed cleavage-embryo transfers of patients with previous repeated failure. Lasers Med Sci. (2019) 34(6):1137–45. 10.1007/s10103-018-02702-3 30627926

[15] Choi KH Lee JH Yang YH Yoon TK Lee DR Lee WS. Efficiency of laser-assisted intracytoplasmic sperm injection in a human assisted reproductive techniques program. Clin Exp Reprod Med. (2011) 38(3):148–52. 10.5653/cerm.2011.38.3.148 22384434 PMC3283067

[16] Debrock S Spiessens C Peeraer K De Loecker P Willemen D D'Hooghe TM. Higher implantation rate using modified quarter laser-assisted zona thinning in repeated implantation failure. Gynecol Obstet Investig. (2009) 67(2):127–33. 10.1159/000171068 19005260

[17] Lee JH Han JE Kim YS Won HJ Cho CH Kwak IP Efficacy of assisted hatching by laser in human IVF-ET program. Clin Exp Reprod Med. (2008) 35:193–202.

[18] Petersen CG Mauri AL Baruffi RL Oliveira JB Massaro FC Elder K Implantation failures: success of assisted hatching with quarter-laser zona thinning. Reprod Biomed Online. (2005) 10(2):224–9. 10.1016/s1472-6483(10)60944-3 15823228

[19] Labarta E Mariani G Holtmann N Celada P Remohí J Bosch E. Low serum progesterone on the day of embryo transfer is associated with a diminished ongoing pregnancy rate in oocyte donation cycles after artificial endometrial preparation: a prospective study. Hum Reprod. (2017) 32(12):2437–42. 10.1093/humrep/dex316 29040638

[20] Mesen TB Mersereau JE Kane JB Steiner AZ. Optimal timing for elective egg freezing. Fertil Steril. (2015) 103(6):1551–6.e1–4. 10.1016/j.fertnstert.2015.03.002 25881876 PMC4457646

[21] Cobo A Garrido N Crespo J José R Pellicer A. Accumulation of oocytes: a new strategy for managing low-responder patients. Reprod Biomed Online. (2012) 24(4):424–32. 10.1016/j.rbmo.2011.12.012 22386762

[22] Dal Prato L Borini A Cattoli M Bonu MA Sciajno R Flamigni C. Endometrial preparation for frozen-thawed embryo transfer with or without pretreatment with gonadotropin-releasing hormone agonist. Fertil Steril. (2002) 77(5):956–60. 10.1016/s0015-0282(02)02960-6 12009350

[23] Shi Y Sun Y Hao C Zhang H Wei D Zhang Y Transfer of fresh versus frozen embryos in ovulatory women. N Engl J Med. (2018) 378(2):126–36. 10.1056/NEJMoa1705334 29320646

[24] Glujovsky D Farquhar C Quinteiro Retamar AM Alvarez Sedo CR Blake D. Cleavage stage versus blastocyst stage embryo transfer in assisted reproductive technology. Cochrane Database Syst Rev. (2016) 6:Cd002118. 10.1002/14651858.CD002118.pub5 27357126

[25] Roque M. Freeze-all policy: is it time for that? J Assist Reprod Genet. (2015) 32(2):171–6. 10.1007/s10815-014-0391-0 25428436 PMC4354191

[26] Melantonio P Attivo J Gomes AP Bonetti TCS Monteleone PAA. Delivering embryos following 10 years of cryopreservation, using unpaired freeze/thaw techniques: a case report. JBRA Assist Reprod. (2021) 25(4):644–6. 10.5935/1518-0557.20210015 34106560 PMC8489820

[27] Bedoschi G Oktay K. Current approach to fertility preservation by embryo cryopreservation. Fertil Steril. (2013) 99(6):1496–502. 10.1016/j.fertnstert.2013.03.020 23535505 PMC3970911

[28] Rienzi L Gracia C Maggiulli R LaBarbera AR Kaser DJ Ubaldi FM Oocyte, embryo and blastocyst cryopreservation in art: systematic review and meta-analysis comparing slow-freezing versus vitrification to produce evidence for the development of global guidance. Hum Reprod Update. (2017) 23(2):139–55. 10.1093/humupd/dmw038 27827818 PMC5850862

[29] Mackens S Santos-Ribeiro S van de Vijver A Racca A Van Landuyt L Tournaye H Frozen embryo transfer: a review on the optimal endometrial preparation and timing. Hum Reprod. (2017) 32(11):2234–42. 10.1093/humrep/dex285 29025055

[30] Grifo JA Hodes-Wertz B Lee HL Amperloquio E Clarke-Williams M Adler A. Single thawed euploid embryo transfer improves IVF pregnancy, miscarriage, and multiple gestation outcomes and has similar implantation rates as egg donation. J Assist Reprod Genet. (2013) 30(2):259–64. 10.1007/s10815-012-9929-1 23307447 PMC3585677

[31] Munné S Wells D Cohen J. Technology requirements for preimplantation genetic diagnosis to improve assisted reproduction outcomes. Fertil Steril. (2010) 94(2):408–30. 10.1016/j.fertnstert.2009.02.091 19409550

[32] Roque M Lattes K Serra S Solà I Geber S Carreras R Fresh embryo transfer versus frozen embryo transfer in *in vitro* fertilization cycles: a systematic review and meta-analysis. Fertil Steril. (2013) 99(1):156–62. 10.1016/j.fertnstert.2012.09.003 23040524

[33] Ribas-Maynou J Novo S Torres M Salas-Huetos A Rovira S Antich M Sperm DNA integrity does play a crucial role for embryo development after ICSI, notably when good-quality oocytes from young donors are used. Biol Res. (2022) 55(1):41. 10.1186/s40659-022-00409-y 36572948 PMC9791757

[34] Alahmar AT Singh R Palani A. Sperm DNA fragmentation in reproductive medicine: a review. J Hum Reprod Sci. (2022) 15(3):206–18. 10.4103/jhrs.jhrs_82_22 36341018 PMC9635374

[35] Solé-Navais P Flatley C Steinthorsdottir V Vaudel M Juodakis J Chen J Genetic effects on the timing of parturition and links to fetal birth weight. Nat Genet. (2023) 55(4):559–67. 10.1038/s41588-023-01343-9 37012456 PMC10101852

[36] Muglia L Tong S Ozanne S Benhalima K. Maternal factors during pregnancy influencing maternal, fetal and childhood outcomes: meet the guest editors. BMC Med. (2022) 20(1):114. 10.1186/s12916-022-02294-4 35264147 PMC8908555

[37] Muglia LJ Benhalima K Tong S Ozanne S. Maternal factors during pregnancy influencing maternal, fetal, and childhood outcomes. BMC Med. (2022) 20(1):418. 10.1186/s12916-022-02632-6 36320027 PMC9623926

[38] Barbitoff YA Tsarev AA Vashukova ES Maksiutenko EM Kovalenko LV Belotserkovtseva LD A data-driven review of the genetic factors of pregnancy complications. Int J Mol Sci. (2020) 21(9):3384. 10.3390/ijms21093384 32403311 PMC7246997

[39] Mardy AH Chetty SP Norton ME. Maternal genetic disorders and fetal development. Prenat Diagn. (2020) 40(9):1056–65. 10.1002/pd.5659 32010984

