# Supplementary material for: Unlocking the potential of embryos: insight of systematic review and meta-analysis into laser-assisted hatching’s role in conquering recurrent implantation failure
Source: Front Reprod Health. 2025 May 9;7:1581529. doi: 10.3389/frph.2025.1581529 (PMC12098289; doi:10.3389/frph.2025.1581529)

**Supplementary Information**

**Supplementary Method 1.** Search strategy for PubMed

**Supplementary Method 2.** Search strategy for Cochrane

**Supplementary Method 3.** Search strategy for Embase

**Supplementary Method 4.** Search strategy for Web of science

**Supplementary Table 1.** Quality assessment of included studies (ROBINS-I tool with kappa coefficient)

**Supplementary Figure 1.** Sensitivity analysis

**Supplementary Figure 2.** Egger’s test for implantation rate.

**Supplementary Figure 3.** Egger’s test for clinical pregnancy rate.

**Supplementary Figure 4.** Egger’s test for abortion rate.

**Supplementary Figure 5.** Egger’s test for term delivery rate.

**Supplementary Figure 6.** Forest plot of implantation rate according to age.

**Supplementary Figure 7.** Forest plot of clinical pregnancy rate according to embryonic status.

**Supplementary Figure 8.** Forest plot of term delivery rate according to embryonic status.

**Supplementary Figure 9.** Forest plot of abortion rate according to embryonic status.

**Supplementary Figure 10.** Forest plot of safty.

**Supplementary Method 1. Search strategy for PubMed**

((Implantation[Title/Abstract]) OR (blastocyst[Title/Abstract]) OR (Embryo[Title/Abstract]) OR (Fertilization in Vitro[MeSH Terms]) OR (In Vitro Fertilization*[Title/Abstract]) OR (Test Tube Fertilization*[Title/Abstract]) OR (Test Tube Bab*[Title/Abstract]) OR (Fertilization, Test-Tube[Title/Abstract]) OR (Fertilizations, Test-Tube[Title/Abstract]) OR (Fertilizations in Vitro[Title/Abstract]) OR (Babies, Test-Tube[Title/Abstract]) OR (Baby, Test-Tube[Title/Abstract]) OR (IVF[Title/Abstract]) OR (Embryo Transfer[MeSH Terms]) OR (Embryo Transfers[Title/Abstract]) OR (Transfer, Embryo[Title/Abstract]) OR (Transfers, Embryo[Title/Abstract]) OR (Blastocyst Transfer[Title/Abstract]) OR (Tubal Embryo Transfer[Title/Abstract]) OR (Tubal Embryo Stage Transfer[Title/Abstract]) OR (ET[Title/Abstract]) OR (Sperm Injections, Intracytoplasmic[MeSH Terms]) OR (Injection, Intracytoplasmic Sperm[Title/Abstract]) OR (Injections, Intracytoplasmic Sperm[Title/Abstract]) OR (Intracytoplasmic Sperm Injection*[Title/Abstract]) OR (Sperm Injection, Intracytoplasmic[Title/Abstract]) OR (Injections, Sperm, Intracytoplasmic[Title/Abstract]) OR (ICSI[Title/Abstract]) OR (repeated embryo implantation failure[Title/Abstract]) OR (recurrent embryo implantation failure[Title/Abstract]) OR (recurrent implantation failure[Title/Abstract]) OR (repeated implantation failure[Title/Abstract]) OR (RIF[Title/Abstract]) OR (failure[Title/Abstract])) AND ((assisted hatching[Title/Abstract]) OR (assisted zona hatching[Title/Abstract]) OR (zona drilling[Title/Abstract]) OR (zona pellucida drilling[Title/Abstract]) OR (zona pellucida opening[Title/Abstract]) OR (zona opening[Title/Abstract]) OR (zona free[Title/Abstract]) OR (zona pellucida dissection[Title/Abstract]) OR (zona pellucida removal[Title/Abstract]) OR (zona pellucida thinning[Title/Abstract]) OR (zona thinning[Title/Abstract]) OR (laser-assisted hatching[Title/Abstract]) OR (laser assisted[Title/Abstract]) OR (laser drilling[Title/Abstract]) OR (laser hatching[Title/Abstract]) OR (laser thinning[Title/Abstract]))

**Supplementary Method 2. Search strategy for Cochrane**

#1 MeSH descriptor: [Fertilization in Vitro] explode all trees

#2 (In Vitro Fertilization):ti,ab,kw OR (In Vitro Fertilizations):ti,ab,kw OR (Test Tube Fertilization):ti,ab,kw OR (Test-Tube Fertilization):ti,ab,kw OR (Test-Tube Fertilizations):ti,ab,kw

#3 (Test Tube Bab):ti,ab,kw OR (Test-Tube Babies):ti,ab,kw OR (Test Tube Babies):ti,ab,kw OR (Test-Tube Baby):ti,ab,kw OR (Fertilization, Test-Tube):ti,ab,kw

#4 (Fertilizations, Test-Tube):ti,ab,kw OR (Fertilizations in Vitro):ti,ab,kw OR (Babies, Test-Tube):ti,ab,kw OR (Baby, Test-Tube):ti,ab,kw OR (IVF):ti,ab,kw

#5 (Fertilization in Vitro):ti,ab,kw OR (IVF-ET):ti,ab,kw

#6 #1 OR #2 OR #3 OR #4 OR #5

#7 MeSH descriptor: [Sperm Injections, Intracytoplasmic] explode all trees

#8 (Sperm Injections, Intracytoplasmic):ti,ab,kw OR (Injection, Intracytoplasmic Sperm):ti,ab,kw OR (Injections, Intracytoplasmic Sperm):ti,ab,kw OR (Intracytoplasmic Sperm Injection*):ti,ab,kw OR (Intracytoplasmic Sperm Injection):ti,ab,kw

#9 (Intracytoplasmic Sperm Injections):ti,ab,kw OR (Sperm Injection, Intracytoplasmic):ti,ab,kw OR (Injections, Sperm, Intracytoplasmic):ti,ab,kw OR (ICSI):ti,ab,kw

#10 #7 OR #8 OR #9

#11 (repeated implantation failure):ti,ab,kw OR (recurrent implantation failure):ti,ab,kw OR (repeated embryo implantation failure):ti,ab,kw OR (recurrent embryo implantation failure):ti,ab,kw OR (RIF):ti,ab,kw

#12 #6 OR #10 OR #11

#13 (assisted hatching):ti,ab,kw OR (assisted zona hatching):ti,ab,kw OR (zona drilling):ti,ab,kw OR (zona pellucida drilling):ti,ab,kw OR (zona pellucida opening):ti,ab,kw

#14 (zona opening):ti,ab,kw OR (zona free):ti,ab,kw OR (zona pellucida dissection):ti,ab,kw OR (zona pellucida removal):ti,ab,kw OR (zona pellucida thinning):ti,ab,kw

#15 (zona thinning):ti,ab,kw OR (laser-assisted hatching):ti,ab,kw OR (laser assisted):ti,ab,kw OR (laser drilling):ti,ab,kw OR (laser hatching):ti,ab,kw

#16 (laser thinning):ti,ab,kw

#17 #13 OR #14 OR #15 OR #16

#18 #12 AND #17

**Supplementary Method 3. Search strategy for Embase**

('laser'/exp OR 'assisted hatching'/exp OR 'assisted zona hatching':ab,ti OR 'zona drilling':ab,ti OR 'zona pellucida drilling':ab,ti OR 'zona pellucida opening':ab,ti OR 'zona opening':ab,ti OR 'zona free':ab,ti OR 'zona pellucida dissection':ab,ti OR 'zona pellucida removal':ab,ti OR 'zona pellucida thinning':ab,ti OR 'zona thinning':ab,ti OR 'laser-assisted hatching':ab,ti OR 'laser assisted':ab,ti OR 'laser drilling':ab,ti OR 'laser hatching':ab,ti OR 'laser thinning':ab,ti) AND ('in vitro fertilization'/exp OR 'fertilization in vitro':ab,ti OR 'in vitro fertilizations':ab,ti OR 'test tube fertilization':ab,ti OR 'test-tube fertilization':ab,ti OR 'test-tube fertilizations':ab,ti OR 'test tube bab':ab,ti OR 'test-tube babies':ab,ti OR 'test tube babies':ab,ti OR 'test-tube baby':ab,ti OR 'fertilization, test-tube':ab,ti OR 'fertilizations, test-tube':ab,ti OR 'fertilizations in vitro':ab,ti OR 'babies, test-tube':ab,ti OR 'baby, test-tube':ab,ti OR 'ivf':ab,ti OR 'embryo transfer'/exp OR 'embryo transfers':ab,ti OR 'transfer, embryo':ab,ti OR 'transfers, embryo':ab,ti OR 'blastocyst transfer':ab,ti OR 'tubal embryo transfer':ab,ti OR 'tubal embryo stage transfer':ab,ti OR 'et':ab,ti OR 'intracytoplasmic sperm injection'/exp OR 'injection, intracytoplasmic sperm':ab,ti OR 'injections, intracytoplasmic sperm':ab,ti OR 'sperm injections, intracytoplasmic':ab,ti OR 'intracytoplasmic sperm injections':ab,ti OR 'sperm injection, intracytoplasmic':ab,ti OR 'injections, sperm, intracytoplasmic':ab,ti OR 'icsi':ab,ti OR 'repeated implantation failure'/exp OR 'recurrent embryo implantation failure' OR 'recurrent implantation failure'/exp OR 'repeated embryo implantation failure' OR 'rif':ab,ti OR 'recurrent embryo implantation failure':ab,ti OR 'repeated implantation failure':ab,ti OR 'recurrent implantation failure':ab,ti OR 'repeated embryo implantation failure':ab,ti OR 'failure':ab,ti)

**Supplementary Method 4. Search strategy for Web of Science**

#1 TS=(Fertilizations in Vitro OR In Vitro Fertilization OR In Vitro Fertilization OR In Vitro Fertilizations OR Test Tube Fertilization OR Test-Tube Fertilization OR Test-Tube Fertilizations OR Test Tube Bab OR Test-Tube Babies OR Test Tube Babies OR Test-Tube Baby OR Fertilization, Test-Tube OR Fertilizations, Test-Tube OR Fertilizations in Vitro OR Babies, Test-Tube OR Baby, Test-Tube OR IVF OR Embryo Transfer OR Embryo Transfers OR Transfer, Embryo OR Transfers, Embryo OR Blastocyst Transfer OR Tubal Embryo Transfer OR Tubal Embryo Stage Transfer OR ET OR Sperm Injections, Intracytoplasmic OR Injection, Intracytoplasmic Sperm OR Injections, Intracytoplasmic Sperm OR Intracytoplasmic Sperm Injection* OR Intracytoplasmic Sperm Injection OR Intracytoplasmic Sperm Injections OR Sperm Injection, Intracytoplasmic OR Injections, Sperm, Intracytoplasmic OR ICSI OR repeated embryo implantation failure OR recurrent embryo implantation failure OR recurrent implantation failure OR repeated implantation failure OR RIF OR failure)

#2 TS=(laser OR laser assisted OR assisted hatching OR assisted zona hatching OR zona drilling OR zona pellucida drilling OR zona pellucida opening OR zona opening OR zona free OR zona pellucida dissection OR zona pellucida removal OR zona pellucida thinning OR zona thinning OR laser-assisted hatching OR laser assisted OR laser drilling OR laser hatching OR laser thinning)

#3 #1 AND #2

**Supplementary Table 1.** Quality assessment of included studies, based on the ROBINS-I tool with kappa coefficient.

| Author,year | Bias due to confounding | Bias in selection of participants into the study | Bias in classification of interventions | Bias due to deviations from intended interventions | Bias due to missing data | Bias in measurement of outcomes | Bias in selection of the reported result | Overall rating |
| --- | --- | --- | --- | --- | --- | --- | --- | --- |
| Petersen 2005 | Low | Low | Low | Low | Low | Low | Low | Moderate |
| Lee 2008 | Low | Low | No imfomation | Low | Low | Low | Low | Low |
| Debrock 2009 | Moderate | Moderate | Low | Low | Low | Low | Low | Moderate |
| Choi 2011 | Low | Low | No imfomation | Low | Low | Low | Low | Low |
| Lu 2016 | Moderate | Low | Moderate | Low | Low | Low | Low | Moderate |
| Artar 2020 | Moderate | Moderate | Low | Low | Low | Low | Low | Moderate |
| Pan 2022 | Moderate | Low | Low | Low | Low | Low | Low | Moderate |
| Curfs 2023 | Low | Low | No imfomation | Low | Low | Low | Low | Low |
| kappa coefficient | 1 | 1 | 0.6 | 1 | 1 | 1 | 1 |  |

**Supplementary Figure 1.** Sensitivity analysis of implantation rate (A); clinical pregnancy rate (B); abortion rate (C); term delivery rate (D) in included articles for the robustness of findings to different aspects of the trials methodology.

**Supplementary Figure 2.** Egger’s test (A), Begg’s test (B) and Labbe plot (C) for implantation rate.

**Supplementary Figure 3.** Egger’s test (A), Begg’s test (B) and Labbe plot (C) for clinical pregnancy rate.

**Supplementary Figure 4.** Egger’s test (A), Begg’s test (B) and Labbe plot (C) for abortion rate.

**Supplementary Figure 5.** Egger’s test (A), Begg’s test (B) and Labbe plot (C) for term delivery rate.

**Supplementary Figure 6.** Forest plot of implantation rateaccording to age.


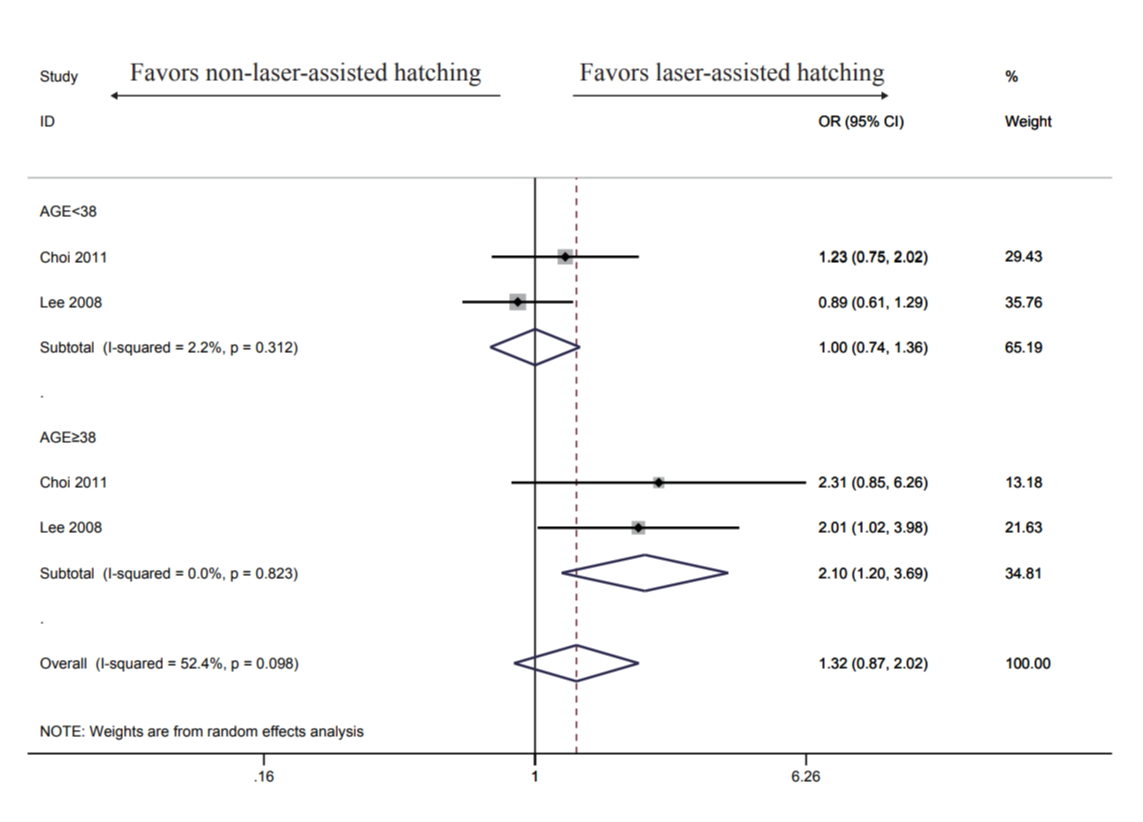


**Supplementary Figure 7.** Forest plot of clinical pregnancy rate according to embryonic status


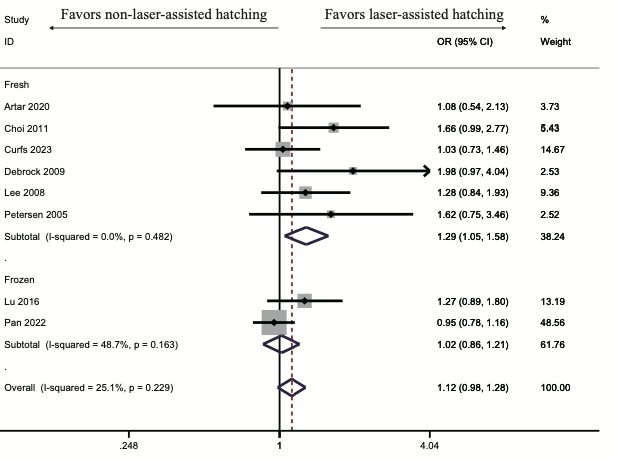


**Supplementary Figure 8.** Forest plot of term delivery rate according to embryonic status.

**Supplementary Figure 9.** Forest plot of abortion rate according to embryonic status.


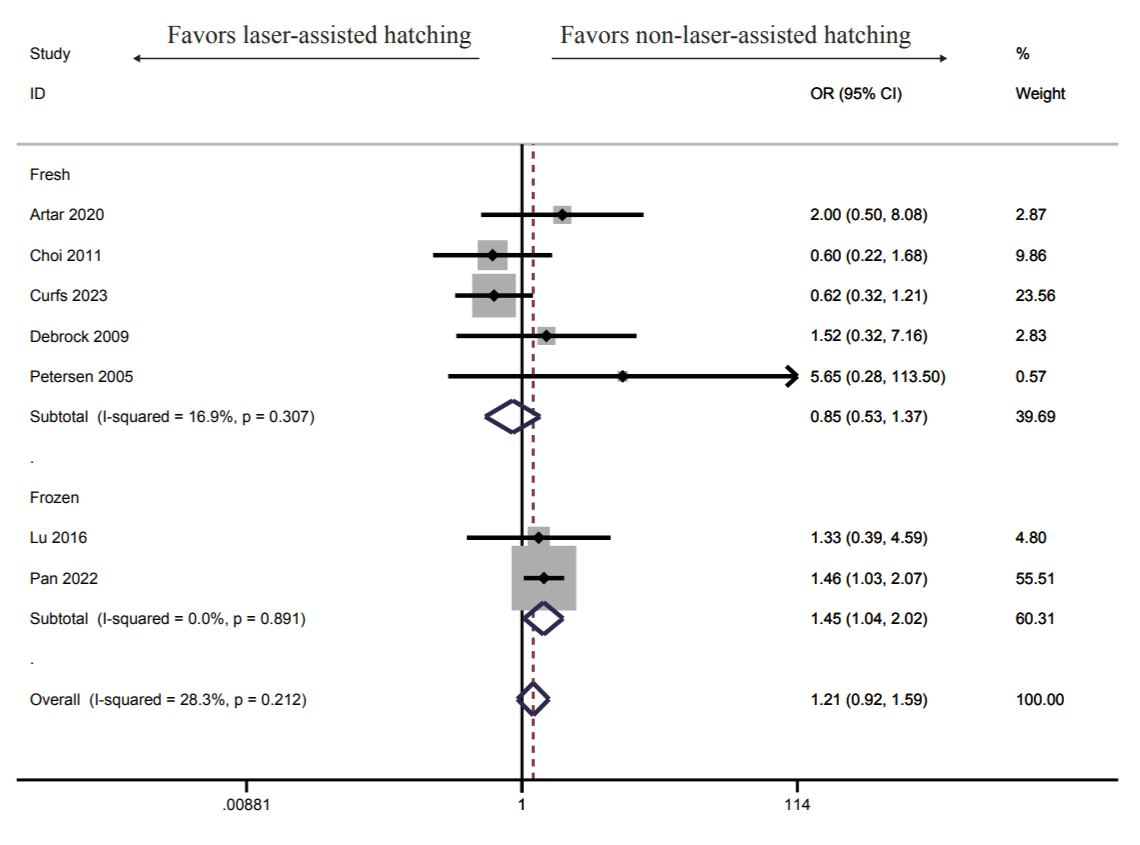


**Supplementary Figure 10.** Forest plot of multiply pregnancy rate (A), ectopic pregnancy rate (B).


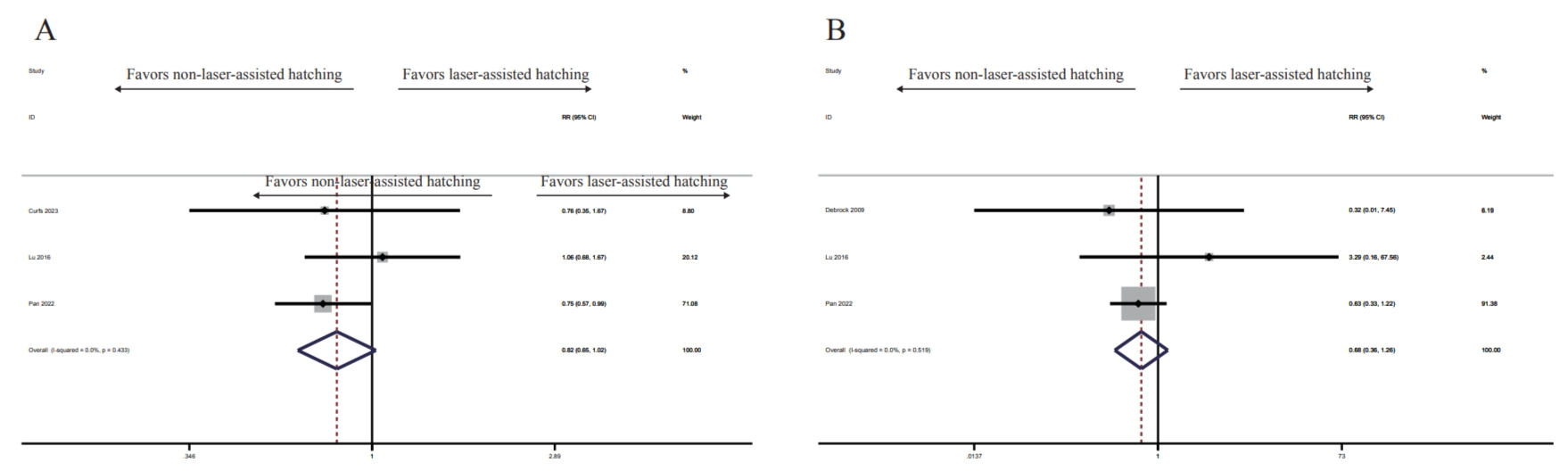

Supplement: Supplementary file 1 [file Datasheet1.docx]
